# The ACTIV-6 Stakeholder Advisory Committee: a model for virtual engagement in decentralized clinical trials

**DOI:** 10.1017/cts.2023.671

**Published:** 2023-11-20

**Authors:** Megan E. Hamm, Jonathan Arnold, Josh Denson, Talethia Edwards, Greg Merritt, Matthew McCarthy, Danielle Nelson, Kirk T. Phillips, Florence Thicklin, Andrew Vasey, Kathleen McTigue

**Affiliations:** 1 Division of General Internal Medicine, University of Pittsburgh, Pittsburgh, PA, USA; 2 Section of Pulmonary, Critical Care, and Environmental Medicine, Tulane University School of Medicine, New Orleans, LA, USA; 3 OneFlorida Clinical Research Network, Gainseville, FL, USA; 4 Patient Is Partner, LLC, Brighton, MI, USA; 5 Department of Medicine, Weill Cornell Medicine, New York, NY, USA; 6 Department of Community Health and Family Medicine, University of Florida College of Medicine, Gainesville, FL, USA; 7 Department of Epidemiology, University of Iowa, Iowa City, IA, USA; 8 CAPriCORN Clinical Research Network, Chicago, IL, USA; 9 Division of General Internal Medicine, University of Nebraska Medical Center, Omaha, NE, USA

**Keywords:** Patient participation, community participation, stakeholder participation, engagement, research design

## Abstract

**Introduction:**

Engaging patients, caregivers, and other stakeholders to help guide the research process is a cornerstone of patient-centered research. Lived expertise may help ensure the relevance of research questions, promote practices that are satisfactory to research participants, improve transparency, and assist with disseminating findings.

**Methods:**

Traditionally engagement has been conducted face-to-face in the local communities in which research operates. Decentralized platform trials pose new challenges for the practice of engagement. We used a remote model for stakeholder engagement, relying on Zoom meetings and blog communications.

**Results:**

Here we describe the approach used for research partnership with patients, caregivers, and clinicians in the planning and oversight of the ACTIV-6 trial and the impact of this work. We also present suggestions for future remote engagement.

**Conclusions:**

The ACTIV-6 experience may inform proposed strategies for future engagement in decentralized trials.

## Introduction to the ACTIV-6 study

The Accelerating COVID-19 Therapeutic Interventions and Vaccines (ACTIV-6) Study is a double-blind, randomized, placebo-controlled, decentralized platform trial studying whether previously FDA-approved medications can be repurposed for the treatment of mild to moderate COVID-19 in the outpatient setting. The study is in the portfolio of the Accelerating COVID-19 Therapeutic Interventions and Vaccines (ACTIV) public/private partnership to identify and address opportunities to address COVID-19 [1]. ACTIV-6 is fully remote and employs site teams at 106 sites across 31 states [2]. Participants are able to complete remote enrollment through their local sites. ACTIV-6 has investigated several potential treatment candidates, and its findings have been widely disseminated [3–6].

To rapidly initiate the ACTIV-6 decentralized clinical trial, NCATS and PCORI executed a Memorandum of Understanding to use the PCORnet infrastructure to collaboratively support site identification and activation for ACTIV-6. Collaboration with PCORnet, which includes the data of >30 million people across the United States, facilitated the potential for diverse recruitment into the trial from all over the United States. Leveraging PCORnet’s emphasis on patient-centered research and tradition of stakeholder engagement [7], each participating PCORnet site was invited to identify up to two stakeholder representatives at study onset. Clinicians (individuals who treat patients with COVID-19) and patients (individuals who have had COVID-19) or caregivers (individuals who have served as a caregiver for someone with COVID-19) were invited to participate in study implementation and guidance.

## Methods

### Structure of our stakeholder engagement

**Figure 1.**
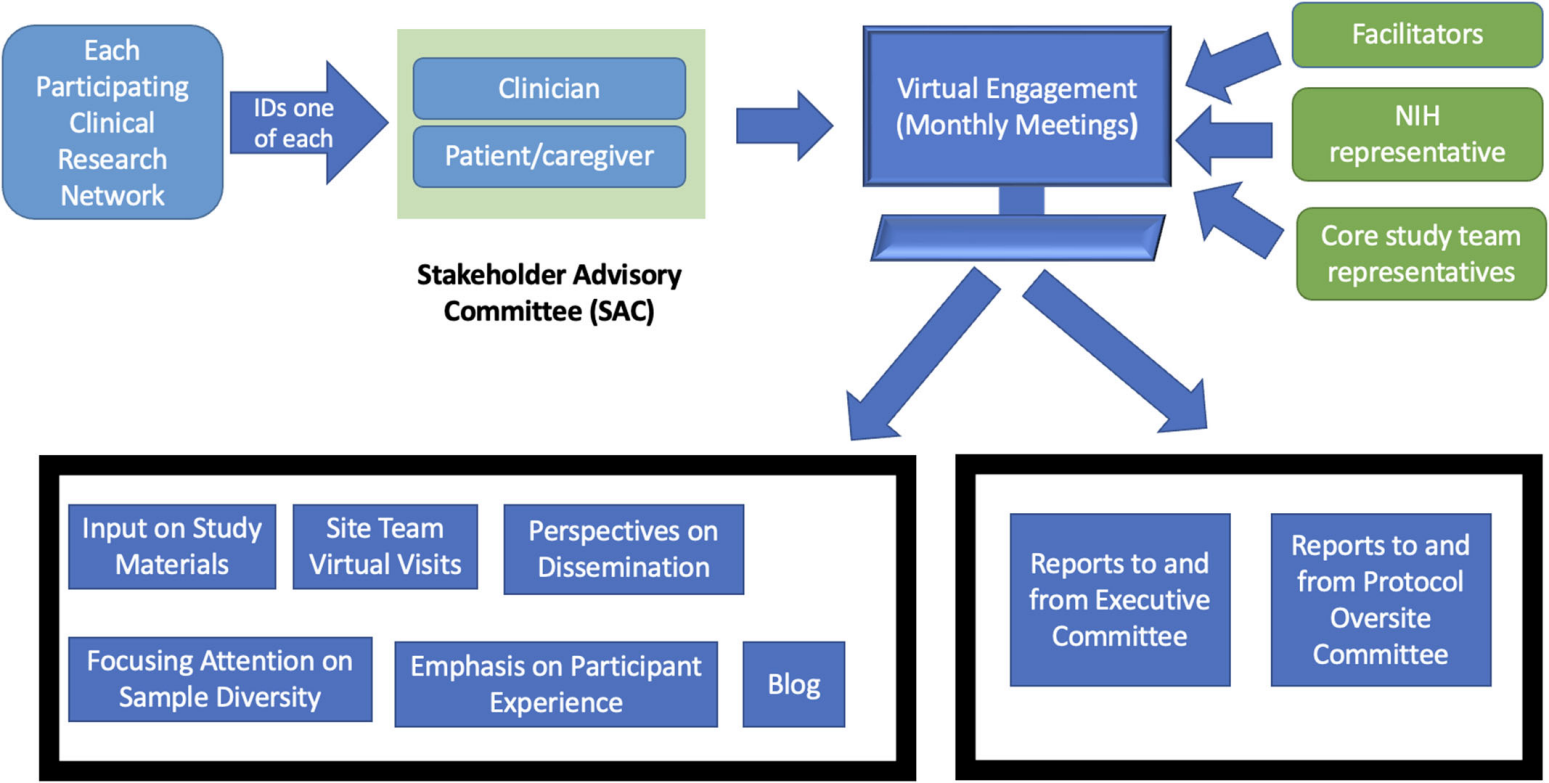
Diagram of ACTIV-6 SAC stakeholder engagement activities and communications.

Research that engages patient and clinician partners in its design, conduct, and dissemination is more likely to generate data that is useful for clinical decision-making, and that is concordant with patient beliefs and needs [8–12]. Our engagement structure began with a well-established core method: *the stakeholder advisory committee* (SAC; Fig. 1) [13–17]. The SAC is five physicians and five patients/caregivers, representing six PCORnet Clinical Research Networks (CAPriCORN, GPC, INSIGHT, OneFlorida, PaTH, and REACHnet). (Note: each of the six clinical research networks invited one clinician and one patient/caregiver, but not all invited chose to participate, resulting in a final SAC membership of 10 individuals.) Recruitment of SAC members from across PCORnet allowed for wide geographic dispersal of selected members and this representation of Americans across much of the country, which was regarded as vital for a pandemic that was, by definition, affecting the entire country. Two SAC facilitators (Megan Hamm and Kathleen McTigue) with engagement expertise as part of the PaTH Clinical Research Network worked to guide the formation of the SAC. Co-leaders representing a patient/caregiver (Ms. Florence Thicklin) and a clinician (Dr. Matthew McCarthy) were chosen by a vote held by all committee members. Ms. Thicklin and Dr. McCarthy served as SAC representatives to the study’s Protocol Oversight Committee and Executive Leadership Committee, thus facilitating SAC coordination with other committees associated with ACTIV-6. For example, SAC members are periodically briefed by Ms. Thicklin and Dr. McCarthy on emerging data patterns and key issues addressed by the study’s Protocol Oversight Committee and Executive Leadership Committee, such as decisions on additional medications to be studied. Conversely, Ms. Thicklin and Dr. McCarthy update the Protocol oversight committee on key issues discussed by the SAC as necessary, such as patient perception of multipurposed medications used in the Study ARMS, and suggested videos that might be developed as part of dissemination of study results to a lay audience.

The SAC was implemented at the beginning of the ACTIV-6 study in April of 2021, when the country was still in the acute phase of the COVID-19 pandemic and vaccines, and antiviral therapies were not yet readily available to the general public. Because of the virtual nature of the trial and the context of the pandemic, SAC meetings were held entirely in Zoom. These meetings were attended by the facilitation team, the SAC co-chairs and members, and representatives from the core ACTIV-6 trial team, the Duke Clinical Research Institute (DCRI), and NIH/NCATS. This combination of attendees allowed for each group – funders, trial scientists, patients and caregivers, and physicians – to learn about the others’ reasoning and perspectives and to come to consensus on components of the trial. Engaging these groups supported an exchange of ideas and perspectives for improving the trial.

SAC activities varied with the course of the trial. Because of the rapidity with which the trial was launched in the context of the pandemic, SAC members could not be involved in the design of the trial and thus confined their ideation and input to trial conduct and dissemination activities. Initial meetings focused on providing training for SAC members regarding stakeholder engagement and patient-partnered research and review of participant-facing trial materials. For example, the SAC reviewed and rewrote draft text for the study website, recruitment flyers, medication packaging inserts explaining the trial, and the informed consent electronic case report form. SAC members used their collective expertise to provide the study team with feedback that informed inclusive participation, a feasible and minimally burdensome participant experience, diverse communications strategies and dissemination methods, and challenges/barriers that may limit participant recruitment and retention. After the trial opened for enrollment, SAC meetings focused on trial updates and recruitment of participants with a particular emphasis on improving participation from under-represented ethnic and racial populations. As ACTIV-6 neared completion, the focus of SAC meetings shifted to plans for effectively disseminating study results to public audiences, including the review of public-facing press releases, continuing diversity recruitment for remaining arms, and appropriately thanking participants.

The SAC used various virtual communication tools to facilitate effective communication between SAC members – who were geographically dispersed as well as from diverse backgrounds, demographics, and types of expertise. For example, the SAC worked with the Clinical Research Network teams who nominated SAC members to arrange virtual video visits between interested SAC members and network-level meeting of the ACTIV-6 clinical site teams recruiting for the trial (Site Team Virtual Visits). These meetings helped SAC members understand the challenges and concerns of their “local” recruitment teams while also providing patient/clinician perspectives to the recruiting teams. In addition, SAC members utilized online surveys to vote on SAC decisions and provide feedback on trial documents. A blog was designed to document SAC activities. It served as a common landing page for SAC members to review decision points, see what had been accomplished, and share their perspectives on the pandemic. The SAC also partnered with the Story Booth project [18], a patient story archive developed and maintained by the PaTH Clinical Research Network, to provide study participants with the opportunity to describe their motivations to participate in the trial and their experiences within ACTIV-6.

## Results

### Impact of the SAC

Throughout the platform trial, SAC contributions were documented via meeting minutes and communications between the SAC and the rest of the study team. SAC contributions fell under four main categories: (1) Changes to study materials; (2) Recommendations on improving patient-centeredness; (3) A focus on diversity in recruitment; and (4) Development of “Thank You” materials and dissemination initiatives. Each of these will be described in more detail below. Quotes representing SAC member thoughts and priorities were selected from meeting transcripts, and are presented in Table 1.

**Table 1.** Stakeholder advisory committee (SAC) impact by area

SAC impact area	Example/quote
Changes to Study Materials Simplify wording Clarify instructions Ensure diversity in images Address unexpected interpretations of written materials	Context: A desire to ensure that medication packaging materials were not unnecessarily frightening to participants given the existing knowledge of the medication. *“Something else that you don't appreciate reading this [a package insert regarding a study drug] is that fluticasone is one of the most commonly prescribed medications in the country, [with] 10s of millions of prescriptions every year in the United States. What I'm reading here makes it sound much more experimental than I think it is. […] I don't know that you [have space on the insert to] include details about just how common and well tolerated this medicine is, but I'm not getting any of that from the language that’s here. It’s prescription medication. […] These are well tolerated potentially effective medicines and what I'm reading here is it just looks like a lot of more scary stuff”*
Recommendations on Improving and Maintaining Patient-Centeredness Focus on ease of use Focus on providing complete information Reminder that participants are ill, and do not feel well, when they are participating – underscoring the need for ease of participation	Context: Review of recruitment and enrollment materials, with a focus on what is said about the study medication, and a desire for more detail. *“I mean, at least as a patient I'd say: I’m not feeling well. You're asking me to do something. I want to know more than what you've just told me [about the medication] that it’s used for other conditions – what conditions [is this medication used for]? I mean that would be my first question.”*
A focus on diversity in recruitment Continued questions about diversity in enrollment Suggestions for recruitment sites and methods	Context: Discussion of need for diversity in recruitment, and suggestions for how to successfully recruit. *Committee Member #1: “I'm not sure we've talked a lot about this before, but it does make me wonder about the who chooses like, how do we ensure that there really will be a truly diverse set of participants involved in this? in part, to learn things like, you know, are there differences amongst folks who take this repurposed drug and show that whether it’s men, women or whether it’s, across racial lines [it’s effective]. So I do think trying to figure out from both the communication in the recruitment side, how do we do this well enough so that we don't end up with a study that that shows a lot of, you know, wealthy white guys.”* *Committee Member #2: You know I think often it’s easy to think that there’s a tool kit we can give to a site or a presentation or a set of sort of talking points, but I think the reality is that the relationship with communities who historically have not been terribly engaged with medical research is not something we can fix with a single training or a single set of tools. I think there’s certainly value in providing some of that, but I think if we really want to increase diversity in this study, I would argue that engaging organizations that are already working with those communities that were interested in and have those relationships are probably going to be more effective, because I think it takes it takes time and more than a single study to build that trust.*
Development of “Thank You” materials and dissemination initiatives Suggestions for how the trial might thank participants Suggestions for how dissemination and thank you activities might create ongoing engagement in research Inclusion of a statement explaining why some adverse events were excluded from the primary analyses for later study arms was added to our CT.gov reporting based on stakeholder feedback	Context: Discussion of terminology to use in thank you’s and dissemination, and the reasons thanking participants is important Committee Member #1: *When you talk about ‘the platform trial’… What does it mean for a study participant? I would be curious to see if an average participant in the study really has any sort of connection to what that means right? Or if they're just in a ‘study’ right?* *Committee Member #2: As volunteers participate in trials, they're undertaking certain kinds of risk and trust. But you know, I come in with Covid and I participated in some trials. But it was a scary time, and a worldwide scary time. So I hope the message to them, however we deliver it, could again embrace an objective of “You volunteered as a trial participant. That’s very important as we advance this science and learn about what works and does not. And you trusted the investigators. So it’s more than a simple ‘Thank You.’ It’s saying: “You took a risk. Look at the benefit that you've brought to our knowledge base and understanding of these medications. And thank you.”*

#### (1) Changes to study materials

Before disseminating any participant-facing materials regarding the ACTIV-6 platform trial, SAC members reviewed documents and provided feedback from their diverse perspectives. This feedback was used by the core research team to iteratively refine these materials, which included recruitment fliers, websites, enrollment forms, study surveys, fact sheets and instructions on taking study medications, and lay-language summaries of trial results. This iterative process was repeated often throughout the operation of the trial. SAC members most often addressed two domains in their feedback:

##### The use of language fitting for a broad participant base

SAC members focused on making sure that language used in participant-facing materials was clear and understandable to community members. They also provided feedback on terminology that might be off-putting. For example, they felt that the use of the word “drug” instead of “medication” might have negative connotations, and all subsequent participant-facing materials used the word “medication.” Additionally, SAC members felt that initial materials regarding medications and their side effects did not adequately represent the known safety and efficacy of these “repurposed” medications in other contexts, and thus made the medications sound more “experimental” than they were. As a result, subsequent materials focused on explaining the extant uses of the medications, and how well-understood study medications already were, while emphasizing the need to take patient safety seriously when using the medications in the novel context of COVID-19 infection.

##### Accessibility of the study for different demographics and geographic areas

SAC members focused feedback on making sure that accompanying imagery featured an array of diverse participants to ensure that it was clear that the trial was welcoming to all. It also ensured that imagery did not represent only one economic group or geographic area in the country. For example, early input on accompanying stock imagery for study materials asked for more and different types of diversity (i.e., age, race, gender, socioeconomic class), and for the possibility of using tailored stock imagery in materials distributed to different communities. The presentation of stick figures in animated/drawn materials, as well, was also reviewed to ensure that the stick figures did not all look identical.

A concrete example of changes to study materials can be found online in Supplement 1, which presents before-and-after ACTIV-6 recruitment flyers, with text summarizing the suggestions made by the SAC and adopted by the broader ACTIV-6 team.

#### (2) Recommendations on improving and maintaining patient-centeredness

Throughout the trial period, SAC recommendations emphasized a need for improving and maintaining patient-centeredness in the trial. These recommendations included viewing all study materials, actions, and requirements through the lens of a potential participant. Because SAC members often had experience with having had COVID-19 themselves, they were able to remind the rest of the study team that potential participants – by definition people in the early stages of infection with COVID-19 who were experiencing symptoms – were feeling unwell at the moment that they considered enrolling, and might be feeling anxious or scared as well. As such, it was important to consider simplicity and ease of participation in all contexts, a concept that was adopted into website designs, simplification of forms, and requests for participant information. SAC feedback of this sort resulted in more streamlined study forms and easier-to-navigate websites, as well as simplification of language. Additionally, SAC members kept the rest of the study team grounded in the sorts of actions a potential participant might take, and their likely outcomes. For example, when preparing materials related to ivermectin, an SAC member did a simple web search for “ivermectin” and found that the first result was an FDA website telling people not to take ivermectin for COVID-19. This emphasized the need for the study to address the controversy over ivermectin with trial participants.

#### (3) A focus on diversity in recruitment

SAC members routinely championed the need to focus on racial, ethnic, geographic, and economic diversity in the trials’ enrolled population. ACTIV-6 sought enrollment in the trial from a patient population mirroring the demographic diversity of the United States. When, at times, actual enrollment in the trial fell behind for some demographic groups, SAC members made suggestions to remedy the issue. Their routine championing of this cause inspired the NIH/NCATS and coordinating center to identify sites excelling in diverse recruitment, identify what they were doing that led to improved diversity in recruitment, and develop and present a series of webinars focused on diversity in recruitment. Study leadership also sought trial sites more likely to recruit or enroll from a diverse population. NIH/NCATS also partnered with the Community Engagement Alliance Consultative Resource (CEACR) team to provide suggestions for enrolling sites to improve diversity. Updates on participant diversity became a standard feature and discussion point of SAC meetings, and the SAC has watched diversity in trial participants (particularly with regards to members of the Hispanic/Latinx community) steadily increase as a result of the combined actions of NIH/NCATS, the trial coordinating center, and local site staff.

#### (4) Development of “thank you” materials and dissemination initiatives

As the trial neared its final year and prepared to close, SAC members were asked to consider how trial participants could be thanked. SAC members brainstormed a variety of ideas, including physical tokens of appreciation such as thank you cards and the production of videos tailored to study participants. Ideas for videos include continuing to focus on dissemination of results in an accessible fashion with a focus on explaining the scientific importance of null findings and the importance of diversity in recruitment for study generalizability. The videos are also each intended as a literal “Thank you” to trial participants in video format. Production of these “Thank you” items is ongoing, and at the time of writing is focused on the production of a public-facing video explaining the study results and what they mean in the current context, including explaining the importance of “null” results, which SAC members indicated might be confusing to community members.

#### (5) Internal evaluation of SAC

Although at the time of writing ACTIV-6 SAC activities are still ongoing, we conducted a survey evaluation of SAC members during September of 2023. Evaluation consisted of a Qualtrics-administered electronic survey utilizing the Ongoing/Long-term Engagement Questionnaire from the Public and Patient Engagement Evaluation Tool (PPEET) [19]. Of the 10 SAC members, 8 completed the survey. Full results, including all open-ended text responses, are presented in Table 2. As can be seen, overall satisfaction with Engagement in ACTIV-6 was high, with SAC members feeling that they had the opportunity to share their viewpoints and positively affect the trial. One survey respondent consistently answered every question as “Strongly Disagree,” but their corresponding open-ended text responses indicated that they were overall very pleased with participation, including the global comment “This experience has been great.” We suspect this respondent made an error in selecting their answers from the scale but have presented the data as collected. While one other member of the SAC did not feel that the study team took SAC feedback into account strongly enough, their open-ended text responses did not provide additional detail into why that was the case. Additionally, comments regarding what could have been improved centered on more communication with the rest of the study team, and occasionally different modes of communication, such as emails and in-person meetings. These responses highlight the importance of study teams incorporating stakeholders' input, and communicating which input is being incorporated in the study results and why, as advised in the following section. Overall, responses indicate that stakeholder experiences were positive and that engagement need not be flawless to be meaningful to stakeholders.

**Table 2.** Full evaluation results. All open-ended text responses are presented in their entirety

	Strongly disagree	Somewhat disagree	Neither agree nor disagree	Somewhat agree	Strongly agree	Total
I have a clear understanding of the purpose of the ACTIV-6 Stakeholder Advisory Committee.	1				7	8
The supports I need to participate in the ACTIV-6 Stakeholder Advisory Committee are available (e.g., travel, childcare)	1				7	8
I have enough information to be able to carry out my role.	1			2	5	8
I am able to express my views freely.	1				7	8
I feel that my views are heard.	1				7	8
A wide range of views on discussion topics is shared.	1			1	6	8
The individuals participating in the ACTIV-6 Stakeholder Advisory Committee represent a broad range of perspectives.	1			1	6	8
This ACTIV-6 Stakeholder Advisory Committee is achieving its stated objectives.	2				6	8
I am confident that ACTIV-6 takes the feedback provided by the ACTIV-6 Stakeholder Advisory Committee into consideration.	2			2	4	8
I think that the work of the ACTIV-6 Stakeholder Advisory Committee makes a difference to the work of the organization.	2				6	8
As a result of my participation in the ACTIV-6 Stakeholder Advisory Committee, I am better informed about COVID-19	1		1	2	4	8
Overall, I am satisfied with this engagement initiative.	1		1		6	8
This engagement initiative is a good use of my time.	1			1	6	8
*Open-ended survey questions*						
What else would you like us to know about how your participation in the ACTIV-6 Stakeholder Advisory Committee is supported?	*“It’s an honor to support this work group and mission*.” (Participant 4) *“ACTIV- 6 stakeholder advisory board has really given me a strong ability to understand COVI-19 and the medicines and studies around it and with the information I gather her I am able to share with my community and providers that are local in my city and state. I have also gained a real interest in medical research and have begun to advocate for African Americans to participate in medical research so that cures and medicines can be effective for us in the future. this group has been sensitive to harsh histories and I am glad to have been apart of such a group.”* (Participant 8)					
What else would you like us to know about how you are able to share your views?	*“All members and staff are engaged and informed.*” (Participant 4) *“Easy to share views. Great that there are voices from all different perspectives.”* (Participant 5)					
In your role, what influence do you think you have had to date?	*“Sharing of data and results to the public.”* (Participant 1) *“I have provided medical expertise to the SAC to explain why we are studying certain drugs, what treatments are currently available, and what future treatments we may need to address the SARS-CoV-2 pandemic.”* (Participant 2) *“Contributed to patient/public informational brochures, website, and clinical journal articles addressing repurposed med’s and related trials.”* (Participant 4) *“I’ve had a voice in the patient facing documents and have helped make sure the message being sent out made sense to anyone viewing it.”* (Participant 5) “Helping to bridge the divide between the medical community and the patient / advocate community. Helping to shape messaging targeting the medical community.” (Participant 6) *“I think my voice has been heard and implemented thus far in how we present this information the community, I have had the opportunity to share and write on the blog as well and inform the team about pushing harder for AA inclusion in the study.”* (Participant 8)					
What else would you like us to know about the influence you think the ACTIV-6 Stakeholder Advisory Committee has had?	“*Translating what is important to the community and patients*.” (Participant 1) *“I would enjoy continuing in this role, and welcome additional opportunities to have an impact. Messaging from public health officials has been challenging, and we should embrace any opportunities to address myths about repurposed med’s.”* (Participant 4) *“I believe we have made the ACTIV-6 project more representative of the community it is attempting to work with, improving the communications and helping to focus recruitment priorities for communities most impacted by COVID.”* (Participant 7) *“I think this committee has had major influenced as I stated earlier in the area of medical research and the need to participate.”* (Participant 8)					
What are the strengths of the ACTIV-6 Stakeholder Advisory Committee?	*“Diverse perspectives, patient centered.”* (Participant 2) *“We have a highly diversified working group, comprised of scientific, clinical, and serving patient/family roles, as well as age, race and ethnic perspectives.”* (Participant 4) *“Wide variety of voices. Everyone is respectful of others.”* (Participant 5) *“Diverse community of participants, open discussions, opportunities for everyone’s voice to be heard.”* (Participant 7) *“The virtual meetings, the sharing of data, the transparency, the willingness to admit hard truths.”* (Participant 8)					
What could be improved about the ACTIV-6 Stakeholder Advisory Committee?	*“More engagement with individual sites that are enrolling patients.”* (Participant 2) “*Expand our opportunities to improve messaging to non-clinical/public audiences. Document our process and prepare for applying our methods to other disease outbreaks, as needed*.” (Participant 4) *“Would be great to have been able to meet in person a time or two. Zoom worked fine though.”* (Participant 5) “*More feedback from the core study team regarding the impact of our work - we have received some of this but could always use more.”* (Participant 7) “I would connect a little more through email in the off times.” (Participant 8)					
What else would you like us to know about your experience with the ACTIV-6 Stakeholder Advisory Committee?	*“Great experience.”* (Participant 2) *“I'm thankful as a patient, to have the opportunity to serve and for the impact that this work may have had.”* (Participant 4) “This experience has been great.” (Participant 8)					

### Strategies for engagement in a distributed platform trial

ACTIV-6 was an entirely virtual trial that was national in scale and sought to address nationwide treatment options for a global pandemic. The scale of the trial and the ubiquity of the questions it sought to answer presented a uniquely challenging environment in which to conduct engagement efforts, which are often very local in scale. Traditional modalities of community engagement – e.g., working with local health advocacy groups or social communities and building relationships and trust over time – do not scale to national engagement in a diverse platform trial but may be necessary to improve recruitment at times in the trial, as noted below. Strategies and lessons learned about patient engagement in such an environment are shared below.

#### Acknowledge the tension between transparency and privacy

Because the ACTIV-6 SAC operated entirely remotely and met once per month, we sought to centralize communications in a public-facing blog that SAC members could check regularly for updates. Initially conceptualized as a place to document SAC actions and share news internally the blog served as a public-facing document that chronicled the advice given by the SAC and resulting actions taken by the trial. This private-and-public-facing nature of the blog raised questions such as: Should the full names of SAC members be available on the blog, or might that constitute a breach of privacy for them? If their complete information was available, might someone from the public contact them about questions related to the trial – and if that happened, what authority and or duty might they have to respond? The SAC mutually decided to use first names only on the blog and to include photos only from SAC members who were comfortable with doing so. Additionally, because SAC members often provided feedback on documents and manuscripts that were not yet available in the public domain, it was not possible to share even highly redacted versions of the meeting minutes in such a public forum without compromising confidential trial information. As a result, blog materials that focused on the existence and general activities were incorporated into the ACTIV-6 website, and the blog itself was ultimately discontinued. When using virtual asynchronous communication in the future, password-protected blogs or remote communication platforms such as Microsoft Teams or Slack as central repositories of SAC communications would ultimately be more viable, with a publicly available blog chronicling curated SAC activities attached.

#### Utilize existing engagement infrastructure

The members of our SAC – a diverse group of individuals from the North East, Midwest, and South – were referred to us through the PCORnet CRNs. Researchers seeking to set up an advisory committee are encouraged to “knock” on the PCORnet Front Door and submit a request indicating that they are interested in finding patient and/or physician partners for their study. Additionally, researchers might find similar help through their local CTSA or other engagement infrastructure available at their institutions.

#### Create and optimize communication structures early

The SAC operated virtually, with committee members dispersed across the country. In the case of ACTIV-6, SAC facilitation is centered in Pittsburgh, PA, and the core trial team is centered in Durham, NC. Trial startup additionally followed an accelerated timeline due to the emergent nature of the pandemic. In such a dispersed environment and amid the acute phase of a global pandemic, routine communication structures between the SAC and the core trial team were not determined at the outset. Successful communication procedures developed organically over time, including the presentations of information by SAC co-chairs Ms. Florence Thicklin and Dr. Matthew McCarthy at other ACTIV-6 committee meetings, and their reporting back to the SAC. It would be beneficial for similar trials in the future to intentionally outline communication structures between the SAC and the rest of the trial team, as well as the intentions behind these communication structures, before the beginning of the SAC’s work. In particular, routine times in executive committee meetings for reporting on SAC activities should be established, as should routine attendance of study PIs or their representatives at SAC meetings. Additionally, training in the rationale for and methods of stakeholder engagement should be offered to the entire study team. Communication channels should be used to highlight which stakeholder input has been adopted to change the overall study, and clearly explain why other input was not adopted.

#### Engage in research reciprocity with SAC members

Research reciprocity is the concept that relationships within research should be reciprocal – i.e., that research participants should be compensated in some way for their participation [20–22]. Although our SAC members are not participants in the trial itself, they are advisors and collaborators on the trial and should also experience reciprocity. SAC members were financially compensated for their time. Future trials and studies should endeavor, as ACTIV-6 did, to compensate SAC members at an hourly rate equivalent to any other specialist or contractor on the grant, or as close to it as possible, although the authors allow that this may not be attainable depending on the project’s funding source. There are, however, additional ways to compensate and show appreciation to stakeholders beyond monetary compensation. For example, ACTIV-6 incorporated research reciprocity into the structure of the SAC by ensuring that the rest of the trial team was responsive to SAC questions and interests. This process began with in-depth training in patient-focused research and stakeholder engagement, which incorporated and tailored training modules developed by PCORI to provide SAC members with better information about their roles in the trial. Then, on a routine basis, the broader study team ensured that the SAC’s scientific questions were answered. For example, representatives from the NIH/NCATS explained questions regarding decisions made by the trial, and physicians on the SAC explained why null results are still exciting and useful. Finally, we engaged experts in topics that SAC members indicated were of interest. For example, following considerable interest by the SAC in improving recruitment of participants from under-represented backgrounds into the trial, the ACTIV-6 team engaged the NIH-funded CEACR team to come speak to the SAC (and the study more broadly) regarding best practices for promoting inclusive participation in clinical research in minority and underserved communities. The NIH/NCATs and DCRI teams also discussed with the SAC the challenges and timelines associated with opening new recruitment sites – particularly sites that may not have established relationships with community organizations.

In addition to the dispersed nature of the trial, the trial itself examined medical treatment for an illness that, in the United States (and across the world) was interpreted differently by different segments of the population, often in politically polarized ways. It also examined a medication, ivermectin, which some physicians and political groups were championing as a “magic bullet” to end the pandemic. Indeed, one of the SAC members’ earliest insights regarded the fact that, when conducting an internet search for “ivermectin,” the first result was a warning from the FDA not to take ivermectin for COVID-19. SAC members argued most people might reasonably conduct such an internet search upon finding out which medication(s) they could be randomized to, and therefore, addressing the reasons this drug was considered safe to take in the context of a clinical trial was thus felt to be of considerable importance for participant trust. The group felt the trial was operating in scientifically important but politically charged waters, posing challenges to communication and acceptance. Because of desire on the part of SAC members to better understand scientific communication in polarized political environments, the SAC moderators identified and invited a Professor of Communications who delivered a talk to the SAC on how to communicate in such an environment. Future trials touching on politically polarizing topics might consider crafting a communication plan around these issues at the outset and training for the study team regarding communicating in polarized environments.

#### Evaluate your engagement

Throughout the trial, we informally sought feedback on the thoughts and feelings of SAC members through meetings and emails aimed at gathering their thoughts on the work as it unfolded. We are planning formal summative engagement activities for the close-out of the SAC at the end of 2023, including surveys and exit interviews assessing SAC and trial team experiences. Because of the rapid start of the trial, and the sense of urgency that trial team members felt at the time, we did not include any elements of formative evaluation. In retrospect, however, we feel that studies can benefit from evaluating their engagement in an ongoing manner throughout the trial and that these evaluation activities need not be burdensome if they consist of simple surveys asking about satisfaction with engagement activities, with more extensive follow-up for surveys indicating any dissatisfaction.

#### Plan for on-the-ground engagement efforts

Throughout our work, we found that a centralized, remote SAC does not replace the need for local engagement work, particularly when it comes to engagement efforts that center on the recruitment of participants. For example, when the SAC suggested additional focus on diversity in recruitment, the work to actually increase diversity in recruitment on the ground had to come through community partnerships and relationships with individual sites. There is a continued need to support and expect local study teams to perform robust community engagement work on behalf of the centralized study team.

### Limitations

As noted above, the rapid startup of the trial in the midst of a national health emergency did not allow for community partner input into the study question. Furthermore, our evaluation of the SAC was conducted prior to the completion of SAC activities and thus did not include evaluation of the perspectives of study members outside the SAC; a broader evaluation utilizing the PPEET Project Questionnaire will be conducted after the SAC concludes its work in December of 2023. Additionally, evaluation did not include trial participants to assess whether or not they felt the trial was patient-centered.

## Conclusions

In conclusion, through the ACTIV-6 trial, we successfully engaged stakeholders from across the country and from many economic, racial, and cultural communities, even in a virtual environment. Stakeholder oversight ensured a focus on the participant experience and a focus on recruitment from the communities most impacted by COVID-19. Due to stakeholder feedback valuable modifications were made in the trial including wording for public informational materials, strategies for maximizing diversity in trial participants, and dissemination of information. This could be a model for future stakeholder advisory groups that are geographically diverse even without the threat of a global pandemic.

## Supporting information

Hamm et al. supplementary material Hamm et al. supplementary material

## Supplementary material

The supplementary material for this article can be found at https://doi.org/10.1017/cts.2023.671
