# Supplementary material for: The ACTIV-6 Stakeholder Advisory Committee: a model for virtual engagement in decentralized clinical trials
Source: J Clin Transl Sci. 2023 Nov 20;7(1):e264. doi: 10.1017/cts.2023.671 (PMC10789984; doi:10.1017/cts.2023.671)
Supplement: Hamm et al. supplementary material [file S2059866123006714sup001.pdf]

SAC members suggested the following changes, which are reflecting in the final flyer.

Imagery

- **Generally positive response to imagery**.
- **Increased diversity** – have options including people of all racial backgrounds, of all age groups and body types.
    - One stakeholder specifically requests "photos that have a multi-generational African American Family (grandparents, parents & children), Latino family, minority group (African American/LatinX in a social setting)"

Wording

- **Generally positive response to wording**. Most stakeholders felt it was clear.
    - One stakeholder suggests the following plain language thesaurus for the study team to use: https://www.plainlanguage.gov/media/Thesaurus_V-10.doc
- **Make sure we consistently use "medication" instead of "drug**."
- **Define placebo the first time it occurs in a document**.
    - A suggested definition, coming from the thesaurus listed above, is "a medication that has no active ingredients and will have no effect."

Layout

- **Layout is generally regarded as crowded**. SAC members suggest that the study team carefully consider what information is needed *at the recruitment stage* vs. what is needed *when enrolling/consenting*.
- **Move inclusion criteria to the top of the flyer** so that they are prominently displayed and those reading the flyer can determine whether they might qualify, and hence whether they should continue reading.
- **Change dots next to inclusion criteria to checkboxes.**
- **Consider removing the following elements from the "What is ACTIV-6?" section to decrease visual clutter on the flyer**:
    - "a worldwide effort to speed the development of treatments and vaccines for COVID-19"
    - The "other goals" section
    - The entire sentence about study medication vs placebo, as this is redundant with information presented elsewhere.

(internal study use only – not available to potential participants)

ACTIV-6

UNDERSTANDING HOW TO HELP PEOPLE WITH COVID-19 FEEL BETTER FASTER

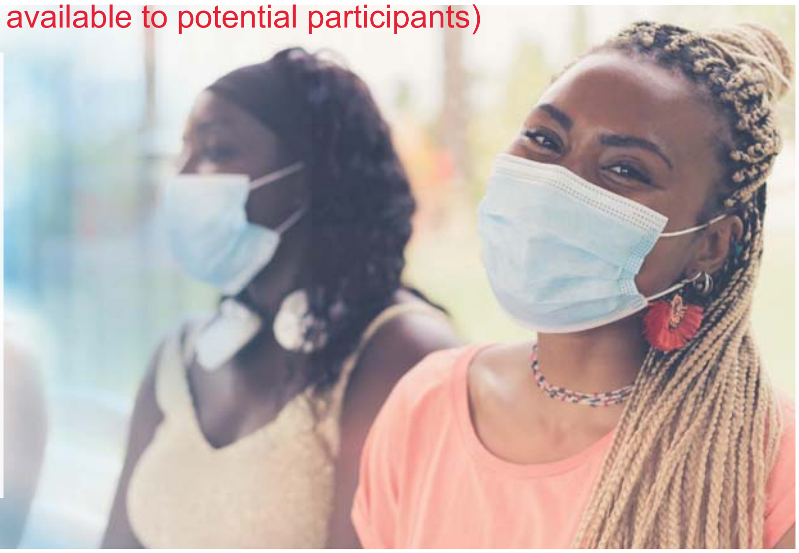

What is ACTIV-6?

ACTIV-6 is part of the Accelerating COVID-19 Therapeutic Interventions and Vaccines (ACTIV) program, a worldwide effort to speed the development of treatments and vaccines for COVID-19.

The purpose of the ACTIV-6 study is to test already-approved drugs to see if they can help people with mild to moderate COVID-19 feel better faster.

Other goals of ACTIV-6 include:

- Finding out if these drugs can lower the risk of more severe disease.
- Learning about the quality of life and long-term symptoms of participants.

Together, we can change the future of COVID-19 treatment.

Some people will take a study drug, while other people will take a placebo. A placebo looks exactly like the study drug but contains no medicine.

Why is this study being done?

Since the COVID-19 pandemic began, progress has been made in treatment and prevention. Vaccines are now available to help prevent infection with the virus that causes COVID-19 (called SARS-CoV-2). However, better treatments for COVID-19 are still needed, especially for patients with mild to moderate illness.

How can you help?

If you answer "yes" to all of these questions, you might be able to take part in this study:

- Are you at least 30 years old?
- Have you tested positive for COVID-19 within the past 10 days?
- Since testing positive, have you had at least 2 symptoms of COVID-19, such as fatigue, trouble breathing, fever, cough, nausea, vomiting, diarrhea, body aches, chills, headache, sore throat, nasal symptoms, or new loss of sense of taste or smell?

Clinical research studies (also called clinical trials) help researchers find out how to treat, prevent, diagnose, and learn about diseases in people. Taking part in clinical research is voluntary and can help improve health care for you and others.

What will you do if you decide to participate?

If you are a good fit for the study and decide to take part, you will:

- Take either a study drug or placebo, as prescribed
- Answer short surveys daily for 14-28 days (no in-person study visits are required)
- Complete a follow-up survey after 90 days

The study drugs will be shipped to you at no cost. You will fill out surveys online. If you have questions, someone will be available to assist you.

We value your contribution! Once the study has ended and results are final, we will share results about how well the study drugs worked among all study participants. Your personal health information is always protected, and individual results are not shared.

TO FIND OUT MORE:
Call:
Email:
Website:
Study site contact:

(publicly available at Help Spread the ACTIV-6 Word | Activ-6 Study (activ6study.org))

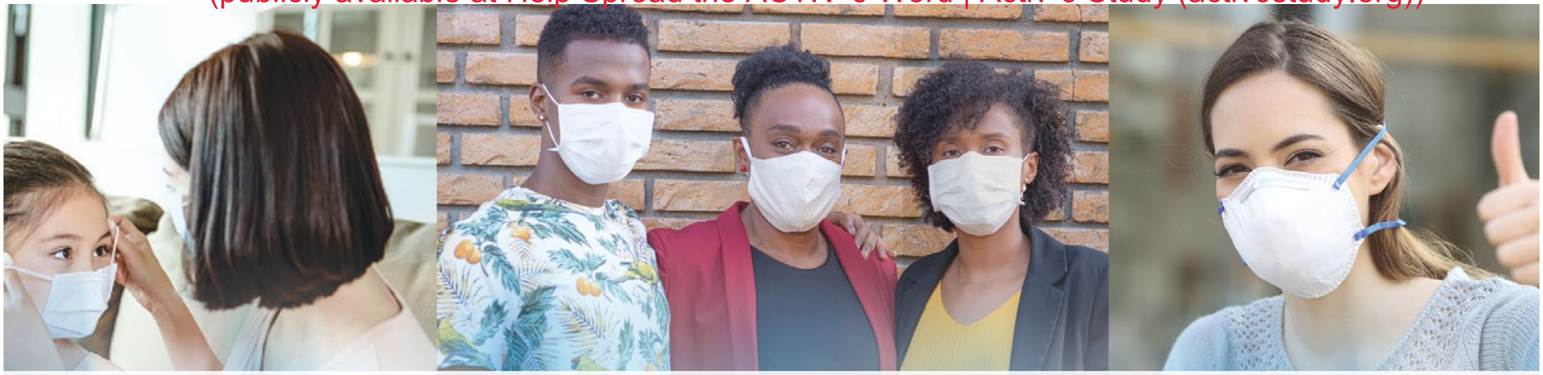

ACTIV-6

WORKING TOGETHER TO HELP PEOPLE WITH COVID-19 FEEL BETTER FASTER.

Together, we can change the future of COVID-19 treatment.

HOW CAN YOU HELP?

If you answer "yes" to all of these questions, you might be able to take part in this study:

- ✔ Are you at least 30 years old?
- ✔ Have you tested positive for COVID-19 within the past 10 days?
- ✔ Are you able to read, speak, and understand English or Spanish?
- ✔ Since testing positive, have you had at least 2 of these COVID-19 symptoms?
    - Feeling tired
    - Trouble breathing
    - Fever
    - Cough
    - Upset stomach
    - Vomiting
    - Diarrhea
    - Body aches or chills
    - Headache
    - Sore throat
    - Stuffy nose
    - New loss of sense of taste or smell

WHAT IS ACTIV-6?

ACTIV-6 is testing medications that are already approved for other diseases to learn if they can help people with mild-to-moderate COVID-19 feel better faster and stay out of the hospital.

ACTIV-6 is part of the Accelerating COVID-19 Therapeutic Interventions and Vaccines (ACTIV) program.

WHY IS THIS STUDY BEING DONE?

Better treatments for COVID-19 are still needed, especially for patients with mild-to-moderate illness. This study is testing different medications because COVID-19 affects each person differently.

ARE COVID-19 TREATMENTS AVAILABLE?

There are now effective treatments that can keep your COVID-19 symptoms from worsening. If your medical provider recommends one of these treatments, you can still choose to participate in ACTIV-6 while taking the treatment.

Please visit combatcovid.hhs.gov to learn more about recommended treatments.

Clinical research studies (also called clinical trials) help researchers learn more about how to treat and prevent diseases and can help improve future health care.

Taking part in ACTIV-6 is voluntary.

You can decide not to participate.

WHAT WILL YOU DO IF YOU DECIDE TO PARTICIPATE?

If you are a good fit for the study and decide to take part, you will:

- Take either a study medication or placebo, as prescribed. A placebo is a medication that has no active ingredients and will have no effect.
- Answer short surveys daily for 14-28 days. Surveys are taken online on a secure, private website. There are no in-person study visits.
- Complete follow-up surveys.

The study medications will be shipped to you at no cost.
Surveys are taken online on a secure, private website.
If you have questions, someone will be available to assist you.

TO FIND OUT MORE

ACTIV6Study.org

Call Center: 833-385-1880
